# Supplementary material for: Prebiotic Galactooligosaccharide Supplementation in Adults with Ulcerative Colitis: Exploring the Impact on Peripheral Blood Gene Expression, Gut Microbiota, and Clinical Symptoms
Source: Nutrients. 2021 Oct 14;13(10):3598. doi: 10.3390/nu13103598 (PMC8537576; doi:10.3390/nu13103598)
Supplement: Supplementary file 1 [file nutrients-13-03598-s001.zip › nutrients-1411387-supplementary.pdf]

Supplemental material

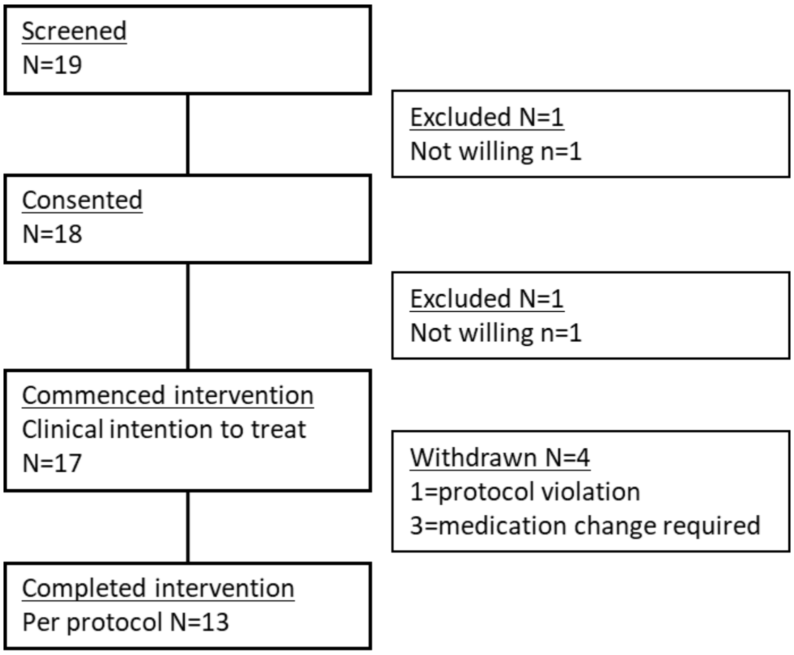

Figure S1: Diagram of patient recruitment to a 6-week open label study of GOS prebiotic in active ulcerative colitis.

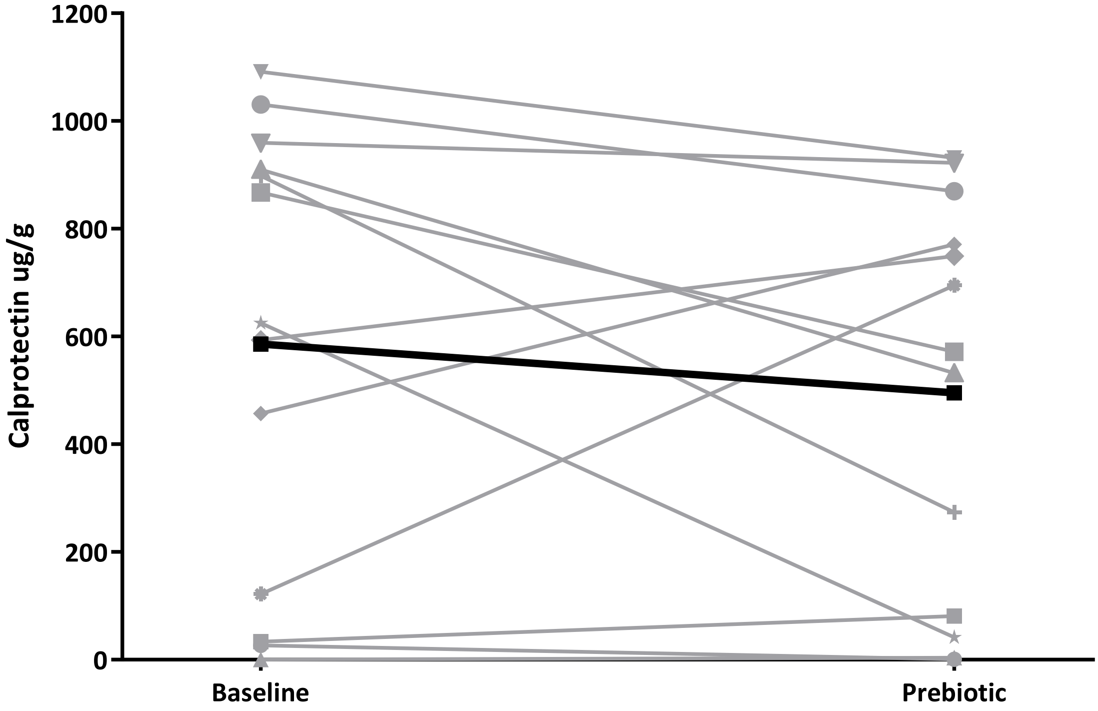

Figure S2: Faecal calprotectin in the PP population (n=13) at baseline and after 6-weeks of prebiotic supplementation in a 6-week open label study of GOS prebiotic in active ulcerative colitis. There were no significant differences between the mean (SD) at baseline (585.71 (415.3)) compared to GOS (495.6 (366.7)) p=0.354. Grey lines indicate individual patient data, black lines indicate the mean values.

Table S1: Demographics and medication of trial participants in a 6-week study of GOS prebiotic in active ulcerative colitis

	Descriptives, n = 17	Minimum-Maximum
Age (years), mean (SD)	35 (10)	24-61
Height (m), mean (SD)	1.73 (0.074)	1.61-1.89
BMI baseline, mean (SD)	25.4 (4.8)	19.8-36.1
BMI 6-weeks, mean (SD)	25.4 (4.9)	19.4-36.4
Female, n (%)	10 (59)	
IBD Medication, n (%)	10 (59)	
5-ASA	10 (59)	
Thiopurine	3 (18)	
Xanthine Oxidase inhibitor	1 (6)	
Other Medication, n (%)	6 (35)	
Antidepressants	2 (12)	
Asthma inhaler	2 (12)	
Other	3 (18)	

Table S2: Faecal short-chain fatty acids and pH in the per protocol population in a 6-week open label study of GOS prebiotic in active ulcerative colitis

	**Per protocol (n=13)**				
**Mean (SD)**	**Baseline**		**Prebiotic**		***p-value**
Total SCFA	2358.1	(1476.5)	2514.4	(1731.4)	0.733
Acetate	1334.9	(878.7)	1496.4	(1060.3)	0.561
Propionate	386.3	(224.5)	391.5	(278.0)	0.954
Butyrate	490.0	(376.4)	459.4	(348.8)	0.687
Valerate	35.7	(25.4)	44.9	(41.0)	0.453
Isobutyrate	47.5	(32.0)	53.6	(44.9)	0.684
Isovalerate	63.7	(52.6)	68.6	(52.6)	0.799
pH	6.4	(0.5)	6.4	(0.5)	0.815

*Data for SCFA are expressed as mg/100g dry weight, *data were compared between baseline and 6-weeks of prebiotic using paired t-tests*

Table S3: Energy and nutrient intake before and after 6-weeks of prebiotic supplementation in a 6-week open label study of GOS prebiotic in active ulcerative colitis

Mean (SD)	Baseline (n=13)		Prebiotic (n=13)		*p
Energy (kcal/d)	2037	(472)	2079	(465)	0.583
Protein (g/d)	89.3	(25.0)	99.4	(27.7)	0.115
Fat (g/d)	90.4	(26.7)	89.1	(29.6)	0.804
Saturated Fat (g/d)	30.1	(10.2)	30.0	(10.5)	0.95
Monounsaturated fat (g/d)	32.9	(12.3)	33.1	(16.0)	0.959
Polyunsaturated fat (g/d)	14.9	(6.2)	13.6	(4.9)	0.237
Transunsaturated fats (g/d)	0.7	(0.4)	0.9	(0.4)	0.161
Carbohydrate (g/d)	201.0	(59.2)	191.0	(54.0)	0.383
Sugars (g/d)	79.3	(30.3)	73.5	(27.5)	0.296
Non starch polysaccharide (Englyst) (g/d)	14.3	(4.7)	12.9	(5.1)	0.165
Fibre (AOAC) (g/d)	19.6	(5.6)	18.2	(6.7)	0.272
Alcohol (g/d)	15.2	(9.7)	29.0	(14.5)	**0.002**
Caffeine (mg/d)	92.5	(53.7)	99.3	(66.1)	0.510

**paired t-test. Data are mean (SD) for energy (kcal/d) and nutrient intake (g/d or mg/d). AOAC, Association of Official Analytical Chemists.*
